# Robert L. Perlman, *Evolution & Medicine*

**DOI:** 10.1093/emph/eou001

**Published:** 2014-01-07

**Authors:** Daniel E.L. Promislow

**Affiliations:** Department of Pathology and Department of Biology University of Washington Box 357705 1959 NE Pacific St. Seattle, WA 98195, USA E-mail: promislo@uw.edu

In 2012, there were 834 769 active physicians in the USA (http://kff.org/other/state-indicator/total-active-physicians/). By my generous and unscientific estimate, I would say that physicians outnumber researchers with a primary interest in evolutionary medicine by more than 1000 to 1. Can this quite small subset of scientists convey the power of evolutionary thinking to the medical establishment? In his new book *Evolution & Medicine*, Robert Perlman sets out to address this important audience.

Evolutionary Medicine is emerging as a vibrant research field of its own, with international meetings devoted to the topic, its own journal, and a steady stream of book-length treatments of the topic. The current excitement in the field owes much to early contributions from George Williams and Randy Nesse, starting with their Quarterly Review of Biology paper almost 25 years ago [1], followed by their book-length treatment of the subject [2], a book now translated into at least nine languages (R. Nesse, personal communication), and cited over 800 times according to Google Scholar. Since then, a variety of books have provided updated introductions to the field as a whole [3–7], whereas others have explored more focused questions (e.g. [8–11]).

These books have many strengths, and I have taught from parts or all of at least six of them. But none of these is directly aimed at the very doctors (or future doctors) whom we would like to influence. Perlman’s book does much to address this shortcoming. The back flap of my copy of *Evolution & Medicine* promises a text that is ‘written for physicians, biomedical scientists, and both premedical and medical students’. The field needs books written for this purpose, and Perlman’s book is a move in the right direction.

This is not to say that previous books are not up to the task. But in the main, they are written either with a broad general audience in mind, or for evolutionary specialists. Perlman recognizes the need to speak directly to physicians and biomedical scientists. He does so right from the start, pointing out that unlike physicians, evolutionary biologists are necessarily focused on variation, the *sine qua non* of natural selection. Each individual is unique, and its phenotype is a reflection of the cumulative outcome of over 3 billion years of evolution acting on its ancestors, coupled with its own recent environmental history. Perlman notes that such variation is decidedly not the purview of the practicing physician:

Medicine has traditionally focused on individuals. Physicians are concerned with the health and well-being of their individual patients. Their primary goal is to keep their patients healthy. When their patients do get sick, physicians are interested in diagnosing their patients’ diseases and in understanding how these diseases cause the symptoms that they do, because they wish to restore their patients to health or at least relieve their discomfort. Only in times of epidemics are physicians concerned with the spread of disease in populations and with ways in which they might help their patients avoid these diseases (p. 7).

From here, Perlman sets out to convince physicians to gently let go of essentialist thinking and to embrace variation. No doctor would deny that human populations are shaped by genetic and environmental variation. Perlman’s goal is to convince them that by understanding this variation, we can better understand why we get sick.

With this thesis in mind, Perlman explores genetic disorders (with a focus on cystic fibrosis), aging, cancer, host–pathogen coevolution (including specialized chapters on STDs and malaria), and finally, some specific diseases associated with human culture, including loss of lactase persistence, diabetes and other diet-associated diseases.

The book is written in a concise manner and the prose is clear, with minimal associated jargon. I would gladly recommend this volume to new medical students and practicing physicians alike, as it will surely help them to recognize the deeper evolutionary explanations behind many human diseases.

Evolutionary medicine recognizes the ubiquity of trade-offs, and Perlman states at the outset that in writing, too, there is an inevitable trade-off between brevity and depth. Perlman favors the former here. However, there are at least three areas where I would have liked to see more extensive treatment.

First, Perlman introduces the reader to the mathematical models used to understand how natural variation evolves (Hardy–Weinberg equilibrium, migration, drift, selection, etc.), and the classic susceptible–infected–recovered models of host–parasite coevolution. But the introduction is equation-free. I would have liked to see equations, and I would have liked to see health-related math problems that challenge the reader to apply those equations. For example, in his thorough discussion of cystic fibrosis, Perlman suggests that the once-beneficial ΔF508 mutation is now always deleterious, noting that ‘it hasn’t yet been reduced to low frequency by natural selection’ (p. 49). Why not give the more assiduous readers the chance to show that lethal recessives can remain at low frequency for an awfully long time?

Second, just as I would like to see visual representations of equations and derivations (even if relegated to text boxes), the book would benefit from more figures throughout (there are just two). It might be a cliché, but a picture really can be worth a thousand words.

And finally, I would have liked to see a more nuanced exploration of some of the more controversial issues in the field. Evolution is a vibrant science, and part of that vibrancy is reflected in the debates. Among the topics that would lend themselves to such discussion are the evolution of virulence (consider [12] versus [13]), and the costs versus benefits of the Paleolithic diet [14].

These are exciting times for evolutionary medicine, as medical schools begin to embrace evolutionary concepts. I hope that we can soon use evolutionary principles not only to explain disease but also to help physicians to prevent and cure disease. To reach this point, physicians must be key players. Books such as this one—aimed squarely at medical students and physicians—will help to bring them into the fold.

## References

[1] Williams GC, Nesse RM (1991). The dawn of Darwinian medicine. Q Rev Biol.

[2] Nesse RM, Williams GC (1994). Why We Get Sick: The New Science of Darwinian Medicine.

[3] Trevathan W, Smith EO, McKenna JJ (1999). Evolutionary Medicine.

[4] Elton S, O’Higgins P (2008). Medicine and Evolution: Current Applications, Future Prospects.

[5] Stearns SC, Koella JC (2008). Evolution in Health and Disease.

[6] Trevathan W, Smith EO, McKenna JJ (2008). Evolutionary Medicine and Health: New Perspectives.

[7] Gluckman PD, Beedle A, Hanson MA (2009). Principles of Evolutionary Medicine.

[8] Ewald PW (1994). Evolution of Infectious Disease.

[9] Diamond JM (1997). Guns, Germs, and Steel: The Fates of Human Societies.

[10] Frank SA (2007). Dynamics of Cancer: Incidence, Inheritance, and Evolution.

[11] Shubin N (2008). Your Inner Fish: A Journey into the 3.5-Billion-Year History of the Human Body.

[12] Ewald PW (1993). The evolution of virulence. Scientific American.

[13] Ebert D, Bull JJ (2003). Challenging the trade-off model for the evolution of virulence: is virulence management feasible?. Trends Microbiol.

[14] Zuk M (2013). Paleofantasy: What Evolution Really Tells Us about Sex, Diet, and How We Live.

